# Mapping heterogeneity in glucose uptake in metastatic melanoma using quantitative ^18^F-FDG PET/CT analysis

**DOI:** 10.1186/s13550-018-0453-x

**Published:** 2018-11-20

**Authors:** Ellen C. de Heer, Adrienne H. Brouwers, Ronald Boellaard, Wim J. Sluiter, Gilles F. H. Diercks, Geke A. P. Hospers, Elisabeth G. E. de Vries, Mathilde Jalving

**Affiliations:** 1 Department of Medical Oncology, University Medical Center Groningen, University of Groningen, Hanzeplein 1, PO Box 30.001, 9700 RB Groningen, The Netherlands; 2 Department of Nuclear Medicine and Molecular Imaging, University Medical Center Groningen, University of Groningen, Groningen, The Netherlands; 3 Department of Pathology, University Medical Center Groningen, University of Groningen, Groningen, The Netherlands

**Keywords:** Stage IV melanoma, ^18^F-FDG PET/CT, Metabolism, SUV, LDH

## Abstract

**Background:**

Metastatic melanoma patients can have durable responses to systemic therapy and even long-term survival. However, a large subgroup of patients does not benefit. Tumour metabolic alterations may well be involved in the efficacy of both targeted and immunotherapy. Knowledge on in vivo tumour glucose uptake and its heterogeneity in metastatic melanoma may aid in upfront patient selection for novel (concomitant) metabolically targeted therapies. The aim of this retrospective study was to provide insight into quantitative ^18^F-fluorodeoxyglucose positron emission tomography/computed tomography (^18^F-FDG PET/CT) parameters and corresponding intra- and inter-patient heterogeneity in tumour ^18^F-FDG uptake among metastatic melanoma patients. Consecutive, newly diagnosed stage IV melanoma patients with a baseline ^18^F-FDG PET/CT scan performed between May 2014 and December 2015 and scheduled to start first-line systemic treatment were included. Volume of interests (VOIs) of all visible tumour lesions were delineated using a gradient-based contour method, and standardized uptake values (SUVs), metabolically active tumour volume (MATV) and total lesion glycolysis (TLG) were determined on a per-lesion and per-patient basis. Differences in quantitative PET parameters were explored between patient categories stratified by *BRAF*^*V600*^ and *RAS* mutational status, baseline serum lactate dehydrogenase (LDH) levels and tumour programmed death-ligand 1 (PD-L1) expression.

**Results:**

In 64 patients, 1143 lesions ≥ 1 ml were delineated. Median number of lesions ≥ 1 ml was 6 (range 0–168), median maximum SUV_peak_ 9.5 (range 0–58), median total MATV 29 ml (range 0–2212) and median total TLG 209 (range 0–16,740). Per-patient analysis revealed considerable intra- and inter-patient heterogeneity. Maximum SUVs, MATV, number of lesions and TLG per patient did not differ when stratifying between *BRAF*^*V600*^ or *RAS* mutational status or PD-L1 expression status, but were higher in the patient group with elevated LDH levels (> 250 U/l) compared to the group with normal LDH levels (*P <* 0.001). A subset of patients with normal LDH levels also showed above median tumour ^18^F-FDG uptake.

**Conclusions:**

Baseline tumour ^18^F-FDG uptake in stage IV melanoma is heterogeneous, independent of mutational status and cannot be fully explained by LDH levels. Further investigation of the prognostic and predictive value of quantitative ^18^F-FDG PET parameters is of interest.

**Electronic supplementary material:**

The online version of this article (10.1186/s13550-018-0453-x) contains supplementary material, which is available to authorized users.

## Background

Novel therapies, especially immunotherapy, have revolutionized the treatment of stage IV metastatic melanoma over the past decade. One-year overall survival (OS) rates have improved to 50–75% and a subset of patients shows durable responses [1]. Still, a considerable number of patients do not respond, especially those with elevated serum lactate dehydrogenase (LDH) levels [1].

The metabolic reprogramming that characterizes cancer cells may well be involved in the efficacy of antitumour immune responses [2, 3]. Cancer cells metabolize a substantial amount of the consumed glucose through glycolysis only—even under aerobic conditions—in order to generate sufficient biomass for rapid cellular proliferation [4, 5]. Novel therapeutic agents interfering with this altered glucose metabolism have shown hints of anticancer activity in (pre)clinical studies, for example in breast cancer, non-small cell lung cancer and glioblastoma [6, 7]. Additionally, preclinical data suggest metabolically targeted therapies can improve antitumour immune response and susceptibility to adjuvant chemo- and radiotherapy [8–10]. In patients, however, such treatments can result in toxicity in highly glucose-dependent healthy tissues, such as the kidney [7, 11]. Furthermore, recent in vitro studies demonstrate that not all melanomas rely on altered glucose metabolic pathways to the same extent [12, 13]. This underlines the need for upfront selection of patients with highly glucose-dependent tumours in order to maximize the benefit of (concomitant) metabolic therapies and ensure a sufficiently broad therapeutic window.

Metastatic melanoma is clinically renowned for its high uptake of the glucose analogue ^18^F-fluorodeoxyglucose (^18^F-FDG) on positron emission tomography/computed tomography (PET/CT) scans. Whole-body ^18^F-FDG PET/CT is therefore part of standard care staging procedures at baseline in stage IV disease, where it is used in a qualitative fashion to provide information on the presence and location of metastases. However, quantitative ^18^F-FDG PET/CT scan analysis has been completely unvisited in stage IV melanoma so far and could provide a wealth of knowledge on quantitative tumour glucose uptake in vivo, potentially useful for upfront patient selection for metabolically targeted therapies. The aim of this retrospective study was to provide an overview of tumour ^18^F-FDG uptake and corresponding intra- and inter-patient heterogeneity in metastatic melanoma patients using quantitative ^18^F-FDG PET/CT scan analysis.

## Patients and methods

### Patients

Patients for this retrospective study were selected from a prospectively maintained database containing all melanoma patients registered at the Department of Medical Oncology of the University Medical Center Groningen (UMCG), the Netherlands, from 2012 onwards. All patients ≥ 18 years of age with histologically proven cutaneous or mucosal metastatic melanoma (American Joint Committee on Cancer [AJCC] 7th edition stage IV melanoma [14]) without prior systemic treatment and with a baseline ^18^F-FDG PET/CT scan performed between May 2014 and December 2015 were eligible for inclusion (*n* = 108). Exclusion criteria were unknown or inadequate adherence to European Association of Nuclear Medicine (EANM) PET/CT scan acquisition guidelines [15] (e.g. PET/CT scan not performed at our hospital) (*n* = 26), no indication for start of first-line systemic treatment within 2 months of baseline PET/CT scan (*n* = 10), concurrent malignancy or other malignancy within the previous 10 years (*n* = 5) and/or no PET-positive lesions (*n* = 3). Ultimately, 64 patients were included (Additional file 1: Figure S1). The Medical Ethics Committee approved the study. Consultation of the local objection registry verified that none of the selected patients had objected to use of their personal data for research purposes. Patients were pseudonymized, and data were stored on a secured server following local data management regulations.

### ^18^F-FDG PET/CT imaging

^18^F-FDG PET/CT scans were acquired using a Siemens Biograph mCT PET/CT system (Siemens/CTI, Knoxville, TN) accredited by the European Association of Nuclear Medicine (EANM) Research Limited (EARL). Scan acquisition and reconstructions were performed following the recommendations of the EANM guideline for oncology ^18^F-FDG imaging [15]. Patients were instructed to fast and avoid exercise at least 4–6 h prior to intravenous ^18^F-FDG injection at an activity of 3 MBq/kg. Serum glucose levels before tracer injection were < 8.3 mmol/l. Whole-body PET/CT scanning (from the top of the skull to the bottom of the feet) was performed 60 min after ^18^F-FDG injection with 1–3 min per bed position. Prior to the PET acquisition, patients underwent a low-dose CT (LD-CT) scan during tidal breathing for attenuation correction (80–140 kVp, quel. ref. 30 mAs and pitch of 1).

### ^18^F-FDG PET/CT scan analysis and volume of interest delineation

All PET/CT scans were initially reported by a nuclear medicine physician as part of routine patient care. Quantitative scan analysis and identification and delineation of all tumour lesions for this study were performed by one investigator (EH) and verified by a board-certified nuclear medicine physician with expertise in melanoma (AB).

PET(/CT) and gradient PET images were displayed side-to-side, and volume of interests (VOIs) were delineated on the gradient PET images using a gradient-based manual contouring method (in-house developed software program). Gradient PET images are derived directly from reconstructed PET images and depict the relative change in counts between neighbouring voxels (Δ standardized uptake value [SUV]), which is typically the highest around tumour borders. Gradient PET images consequently provide an image where the borders of the lesion are most intense. Use of gradient PET data enables a (manual) VOI delineation method where lesion border location is independent of colour scale, in contrast to manual contouring on regular PET images. Additional motives for choosing gradient-based delineation were a lack of systematic delineation studies in metastatic melanoma and inaccuracy of EARL-recommended semi-automatic delineation methods for delineation of large heterogeneous tumour lesions or small yet highly ^18^F-FDG-avid lesions [15].

A region of interest (ROI) was manually drawn around each tumour lesion on consecutive transaxial slices. Subsequently, the observer adjusted a %-threshold based on maximum SUV (SUV_max_) until the VOI borders optimally corresponded with the location of the steepest gradient on the gradient PET images as judged visually. SUV_max_, mean SUV (SUV_mean_), peak SUV (SUV_peak_, i.e. a 1.2-cm^3^ spheric region positioned to yield the highest average value), metabolically active tumour volume (MATV) and total lesion glycolysis (TLG, the product of SUV_mean_ and MATV) were determined for each VOI. SUVs were corrected for serum glucose level and lean body mass according to the Janmahasatian formula [15].

Lesions with an MATV < 1 ml were excluded from the final quantitative analysis to prevent partial volume effects. PET parameters were analysed on a per-patient, per-location and per-lesion basis. Patient’s maximum SUV and median SUV reflect respectively the highest and median value derived from all lesions ≥ 1 ml within that patient. Interquartile range (IQR) SUV_peak_ was derived from the SUV_peak_s of all individual lesions delineated in one patient as a measure for intra-patient ^18^F-FDG uptake heterogeneity. Total MATV or total TLG equals the sum of respectively MATV or TLG of all lesions ≥ 1 ml within that patient.

### CT and brain MRI scan analysis

Previously, PET-negative (i.e. with SUV_max_ < 1.5) melanoma metastases have been described, and we excluded three eligible patients upfront due to the presence of only PET-negative lesions [16]. Therefore, we aimed to evaluate the first 20 included patients for the presence of PET-negative lesions with a diameter ≥ 1 cm on baseline contrast-enhanced CT (ce-CT) scan performed within 1 month of the baseline PET/CT. ce-CT scan was available in 12 of the 20 patients and revealed only 2 additional ^18^F-FDG PET-negative lesions ≥ 1 cm on top of the total of 491 PET-positive lesions > 1 ml in these patients (0.4%). Due to this limited additional value, ce-CT analysis was omitted for the remaining patients.

High physiological background ^18^F-FDG uptake prevents accurate detection and quantification of brain metastases. Therefore, the presence of brain lesions was additionally evaluated on baseline cerebral MRI scans or cerebral ce-CT. Quantitative data from brain lesions were not incorporated in per-patient PET parameters. When brain lesions were measurable (longest axis on MRI > 1 cm according to Response Assessment in Neuro-Oncology Brain Metastases [RANO-BM] criteria [17]) and ^18^F-FDG-avid, SUV_peak_ and SUV_max_ were measured.

### Data acquisition

Patient and tumour characteristics, baseline serum LDH levels and respectively tumour *BRAF* and *RAS* mutation status were retrospectively determined from the electronic patient file. Pre-treatment serum LDH levels were derived from the date closest to the baseline PET/CT scan. When pre-treatment archival tumour biopsies for a distant metastasis were available, PD-L1 immunohistochemistry (IHC) was performed as previously described elsewhere using the 22C3 anti-PD-L1 antibody (DAKO, Merck) on Ventana BenchMark ULTRA platform [18]. Tissue derived from primary melanomas, local recurrences, in-transit cutaneous metastases or lymph node metastases was excluded. Scoring was performed by two board-certified pathologists (GFHD, NAH) and performed according to the manufacturer’s instructions.

### Statistical analysis

Variables were assessed for normal distribution by Q-Q plots. Independent Mann-Whitney *U* tests were used to assess differences in PET parameters between LDH, *BRAF* and *RAS* groups, respectively, and Kruskal-Wallis tests for differences between metastatic locations and PD-L1 expression groups. Spearman’s rank correlation was used for the correlation between lesion MATV and SUV_peak_. A *P* value < 0.05 (two-sided) was considered statistically significant. Statistical analysis was performed using SPSS Statistics, version 23.0 (IBM Corp., Armonk, NY).

## Results

### PET parameters on a per-patient basis

**Fig. 1:**
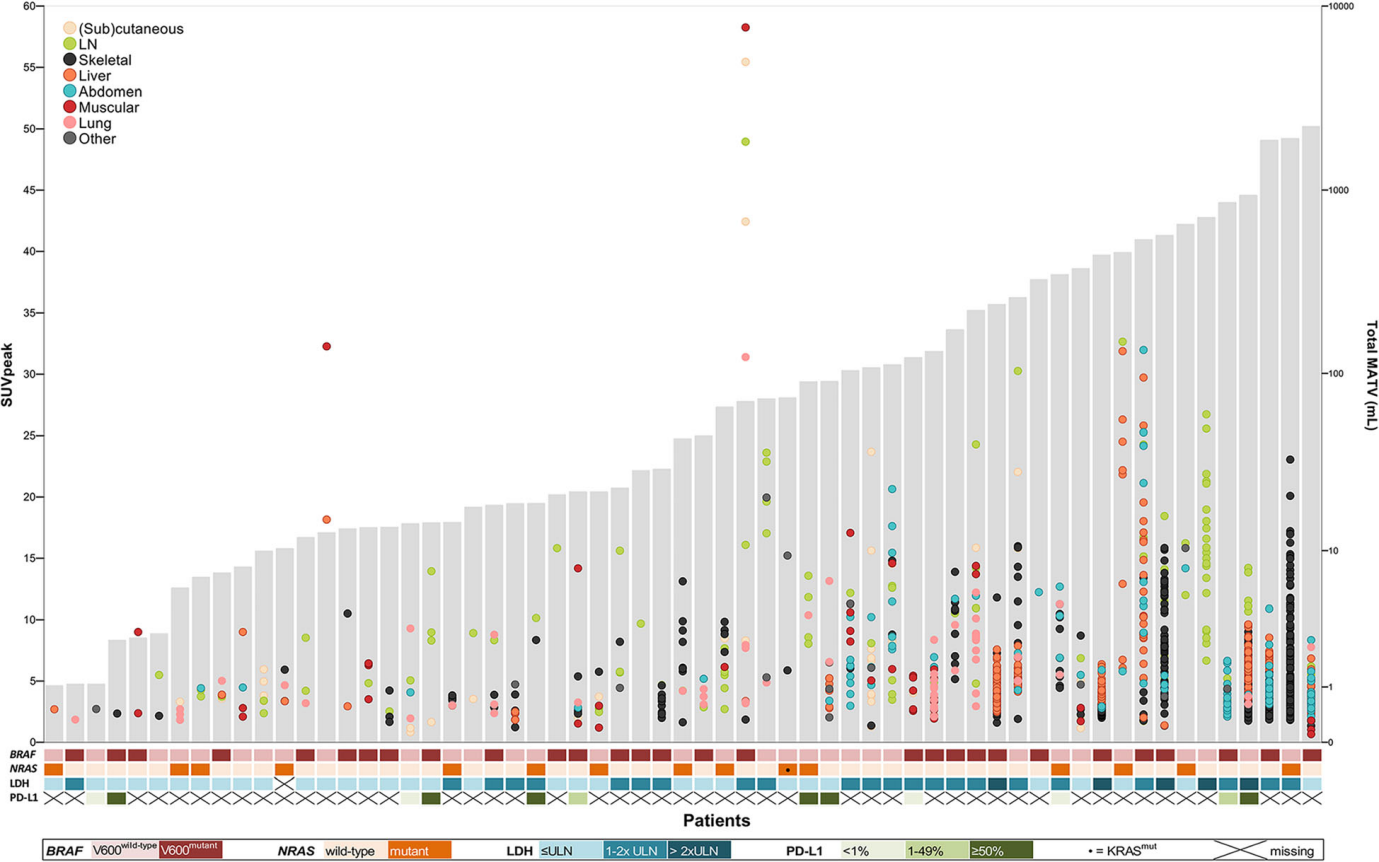
Individual tumour lesions (≥ 1 ml) and their SUV_peak_ displayed per patient. For each patient (*x*-axis; *n* = 64), individual tumour lesions are plotted against their SUV_peak_ (left *y*-axis). Grey shaded bars represent the patient’s total MATV (right *y*-axis). The heatmap displays respectively the patient’s LDH level and tumour *BRAF* and *NRAS* status and PD-L1 expression. Three patients are not displayed since they only had lesions < 1 ml, which resulted in SUVs, a MATV and TLG of 0. *LDH* lactate dehydrogenase, *LN* lymph node, *PD-L1* programmed death-ligand 1, *ULN* upper limit of normal

Patient characteristics are presented in Table 1. *BRAF*^*V600*^ mutational status did not differ between patients with normal or elevated serum LDH (42.9% vs. 57.1%; *P =* 0.260)*.* Patient’s maximum SUV_peak_ showed a broad range (0–58; median 9.5) between patients (Fig. 1 and Table 2). Furthermore, intra-patient ^18^F-FDG uptake heterogeneity was observed, with SUV_peak_ IQR ranging from 0 to 42.4 (median 2.1). The number of lesions, SUVs, total MATV and total TLG (per-patient basis) did not differ between *BRAF*^V600^ mutant vs. wild-type patients (Table 3), *RAS* mutated vs. wild-type patients and *BRAF*^V600^/*RAS* mutant vs. *BRAF*^*V600*^*+RAS* wild-type patients (data not shown). Patients with an elevated LDH level (> 250 U/l) had more lesions ≥ 1 ml (median 17 vs. 4, *P* < 0.001), a higher total MATV (127 vs. 14 ml, *P* < 0.001), higher maximum SUV_peak_ (13.3 vs. 8.7, *P* = 0.011), SUV_max_ (15.8 vs. 11.3, *P* = 0.026) and SUV_mean_ (9.0 vs. 6.0, *P* = 0.009) and higher total TLG (1180 vs. 67, *P* < 0.001) (Table 3). Of the 13 tumour specimens that were available for PD-L1 IHC, 4 showed < 1% PD-L1 expression, 3 1–49% and 6 ≥ 50%. PD-L1 expression status did not correlate with any of the PET parameters (data not shown).

**Table 1:** Patient characteristics

Characteristic	All patients (*n* = 64)
Gender	
Male	40 (62.5%)
Female	24 (37.5%)
Age (years) at baseline PET/CT	59 (45–69) (range 25–80)
World Health Organization performance	
0	45 (70.3%)
1	7 (10.9%)
≥ 2	7 (11.0%)
Missing	5 (7.8%)
Histology primary melanoma	
Cutaneous	47 (73.4%)
Mucosal	4 (6.3%)
Primary melanoma unknown/missing	13 (20.3%)
M-stage at baseline PET/CT	
M1a	1 (1.6%)
M1b	2 (3.1%)
M1c	61 (95.3%)
No. of different metastatic locations^a^	
1	3 (4.7%)
2	6 (9.4%)
> 2	55 (85.9%)
Organ involvement	
(Sub)cutaneous	39 (60.9%)
Lymph nodes	54 (84.4%)
Lungs	40 (62.5%)
Muscular	25 (39.1%)
Skeletal	39 (60.9%)
Liver	24 (37.5%)
Abdomen^b^	30 (46.9%)
Other^c^	14 (21.9%)
Brain metastases^d^	
Yes	22 (34.4%)
^18^F-FDG-avid^e^	11 (17.2%)
Not ^18^F-FDG-avid	11 (17.2%)
No	35 (54.7%)
Missing	7 (10.9%)
*BRAF* mutation status	
*BRAF*^V600^ mutation	31 (48.4%)
No *BRAF*^V600^ mutation	33 (51.6%)
*RAS* mutation status	
*RAS* mutation^f^	15 (23.4%)
No *RAS* mutation	49 (76.6%)
Baseline serum LDH (U/l)	246 (192–327) (range 92–11,371)
Normal	35 (54.7%)
Elevated^g^	28 (43.7%)
> 1–2× ULN	23 (35.9%)
> 2× ULN	5 (7.8%)
Missing	1 (1.6%)
Interval between baseline PET/CT and LDH measurement (days)	0 (− 7 to + 3) (range − 39 to + 11)

Data are displayed as *n* (%) or median (interquartile range)

*LDH* lactate dehydrogenase, *ULN* upper limit of normal

^a^Including brain metastases

^b^Number of patients with lesions in the abdominal cavity/peritoneum (*n* = 27; 42.2% of all patients), adrenal gland (*n* = 12; 18.8%), bowel (*n* = 6; 9.4%), spleen (*n* = 3; 4.7%), kidney (*n* = 2; 3.1%), gallbladder (*n* = 1; 1.6%), stomach (*n* = 1; 1.6%), rectum (*n* = 1; 1.6%) and/or pancreas (*n* = 1; 1.6%)

^c^Number of patients with lesions in the vaginal or nasal mucosa (*n* = 4; 6.3%), myelum (*n* = 1; 1.6%), shoulder joint (*n* = 2; 3.1%), breast (*n* = 2; 3.1%), pericardium (*n* = 3; 4.7%), heart (*n* = 2; 3.1%) and/or abdominal or thoracic wall of undetermined tissue of origin (*n* = 2; 3.1%)

^d^Based on MRI brain (*n* = 53) or, when missing, contrast enhanced CT (*n* = 4)

^e^I.e. distinguishable from normal brain tissue

^f^*NRAS* (*n* = 14) and *KRAS* (*n* = 1)

^g^I.e. > 250 U/l

**Table 2:** ^18^F-FDG PET tumour lesion parameters on a per-patient basis

	All patients (*n* = 64)	Range
No. of lesions		
All	18 (11–51)	1–417
≥ 1 ml^a,b^	6 (2–16)	0–168
SUV_peak_		
Maximum	9.5 (5.5–15.5)	0–58.3
Median	4.3 (3.2–8.6)	0–25.2
SUV_peak_ interquartile range^c^	2.1 (0–5.1)	0–42.4
SUV_max_		
Maximum	11.8 (7.3–18.0)	0–67.2
Median	6.1 (4.1–11.5)	0–37.6
SUV_mean_		
Maximum	7.2 (4.7–10.7)	0–30.2
Median	4.3 (3.0–7.3)	0–18.5
Total MATV (ml)	29.2 (12.2–234)	0–2212
Total TLG	209 (46.2–1510)	0–16,740

Data are displayed as median (interquartile range)

^a^Three patients had only lesions < 1 ml

^b^I.e. all lesions included in quantitative analyses

^c^I.e. interquartile range of the different SUV_peak_s measured within one patient, measure of intra-patient heterogeneity

**Table 3:** ^18^F-FDG PET lesion parameters on a per-patient basis, stratified by LDH or *BRAF*^*V600*^ mutation status

	LDH groups^a^		*P*	*BRAF*^*V600*^ groups		*P*
	Normal^b^ (*n* = 35)	Elevated (*n* = 28)		Wild-type (*n* = 33)	Mutant (*n* = 31)	
No. of lesions						
All	13 (7–17)	46 (20–140)	< 0.001	17 (10–34)	18 (11–64)	0.510
≥ 1 ml^c^	4 (2–6)	17 (7–48)	< 0.001	6 (3–14)	6 (2–26)	0.984
SUV_peak_						
Maximum	8.7 (4.4–13.1)	13.3 (7.1–23.5)	0.011	10.1 (5.6–18.9)	8.8 (5.2–13.9)	0.310
Median	3.9 (2.7–7.4)	5.5 (3.5–8.9)	0.203	5.3 (3.3–9.0)	4.0 (3.2–8.3)	0.317
SUV_peak_ interquartile range^d^	1.2 (0–3.3)	3.3 (1.5–6.7)	0.002	2.7 (0–5.1)	2.0 (0–4.7)	0.380
SUV_max_						
Maximum	11.3 (6.1–16.6)	15.8 (9.0–27.7)	0.026	13.0 (7.3–22.2)	11.6 (7.2–17.4)	0.344
Median	5.3 (3.7–9.4)	8.0 (4.9–12.0)	0.171	7.4 (4.5–11.7)	5.3 (4.0–11.7)	0.394
SUV_mean_						
Maximum	6.0 (4.1–8.7)	9.0 (6.1–15.4)	0.009	8.4 (4.7–12.3)	6.9 (4.7–8.8)	0.274
Median	3.9 (2.8–6.2)	5.3 (3.4–7.6)	0.128	4.8 (3.2–7.9)	3.9 (2.9–7.1)	0.256
Total MATV (ml)	14 (6–65)	127 (29–512)	< 0.001	44 (10–185)	29 (13–238)	0.861
Total TLG	67 (18–448)	1180 (200–2998)	< 0.001	281 (43–1541)	199 (64–1568)	0.984

Data are displayed as median (interquartile range)

*LDH* lactate dehydrogenase

^a^One patient had a missing LDH value

^b^Three patients with normal LDH had only lesions < 1 ml

^c^All lesions included in subsequent quantitative analyses

^d^Interquartile range of the different SUV_peak_s measured within one patient, measure of intra-patient heterogeneity

### PET parameters on a per-lesion and per-location basis

In total, 3408 tumour lesions were delineated, of which 1143 had an MATV ≥ 1 ml. Median lesion SUV_peak_ was 5.0 (range 0–58), median MATV was 2.4 ml (range 1.0–1921) and median TLG 11 (range 1.1–11,206) (Additional file 2: Table S1). Lesion SUV_peak_ and MATV were moderately correlated (correlation coefficient 0.521, *P* < 0.001). The highest numbers of separate lesions were observed in bone (*n* = 504, 44% of all lesions ≥ 1 ml), liver (*n* = 241, 21%) and lymph nodes (*n* = 125, 11%) (Additional file 3: Figure S2a), and total measured MATV was highest in the abdomen (5683 ml, 39%) followed by bone (3864 ml, 27%) and lymph nodes (2321 ml, 16%) (Additional file 3: Figure S2b). No major differences between metastatic locations concerning individual lesion’s MATV and SUV_peak_ were observed (Additional file 4: Figure S3).

Brain metastases were present in 22 patients, and 16 had measurable disease according to RANO-BM criteria [17]. In 11 of these patients, brain metastases were visible as hypermetabolic lesions on ^18^F-FDG PET/CT. Median SUV_peak_ and SUV_max_ of these lesions were 7.2 (range 4.8–36.8) and 9.0 (6.5–45.4), respectively.

### Overall survival

Following the baseline PET/CT scan, patients commenced standard systemic treatment consisting of either immune checkpoint inhibition, BRAF(/MEK) inhibition and/or dacarbazine chemotherapy. Given the various systemic treatments used, overall survival analysis was performed for exploratory purposes only (Kaplan-Meier overall survival curves in Additional file 5: Figure S4).

## Discussion

We show major intra- and inter-patient heterogeneity in tumour lesion ^18^F-FDG uptake among metastatic melanoma patients. Presence of tumours with above median ^18^F-FDG uptake was independent of tumour mutational status and did not fully coincide with high serum LDH level. This suggests that tumour ^18^F-FDG uptake is an independent feature and that ^18^F-FDG PET parameters might be suitable as a selection tool for novel metabolic therapies.

This is the first large study providing an overview of intra- and inter-patient differences in tumour glucose consumption in metastatic melanoma patients using quantitative whole-body imaging of ^18^F-FDG uptake. Previous melanoma studies on ^18^F-FDG PET/CT imaging focused on its diagnostic accuracy for qualitative lesion detection and/or used quantitative parameters derived from the primary melanoma or only a limited number of (the most intense) lesions for response evaluation or prognostic models. By performing quantitative evaluation of all tumour lesions, we highlight the utility of ^18^F-FDG PET/CT in demonstrating heterogeneity of glucose uptake among metastatic melanoma patients. Preliminary estimates of the influence of tumour ^18^F-FDG uptake on survival support further prospective investigation as a prognostic biomarker.

Compared to previous studies, we found a higher proportion of bone metastases and a lower incidence of lung and soft tissue metastases. Two previous studies in metastatic melanoma using ^18^F-FDG PET/CT ± other imaging methods qualitatively report metastases predominantly to the lung, liver, lymph nodes and skin/soft tissue [19, 20]. This difference might be explained by differing patient populations, especially since our study also included patients with an unknown primary melanoma and subsequent widespread (skeletal) metastases (*n* = 8), as opposed to the study performed by Schoenewolf et al. [19]. Furthermore, we excluded lesions with an MATV <  1 ml to minimize partial volume effects. Three patients had only lesions with an MATV < 1 ml, which all concerned metastases in the lymph nodes, lung, subcutis and/or muscles. The small MATV at these locations, resulting in the exclusion of these lesions for the analysis, further explains the smaller fraction of soft tissue, lymph node and subcutaneous lesions in our PET-based study.

*BRAF*^*V600*^ mutant melanoma cells rely heavily on glycolysis with high glycolytic rates induced by activation of the mitogen-activated protein kinase (MAPK) pathway [21, 22]. *BRAF*^*V600*^ wild-type melanomas (approximately 50% of melanomas) often have alternative mutations in the MAPK-pathway including *RAS* or *MEK1/2* that are also associated with glycolytic dependency and increased glucose uptake [23–25]. In thyroid carcinoma, *BRAF*^*V600E*^ tumours show increased expression of glucose transporter (GLUT) and higher SUVs compared to *BRAF*^*V600*^ wild-type tumours [26, 27]. We found no difference in tumour glucose uptake and MATV between patients with and without a *BRAF*^*V600*^ or *RAS* mutation. Overexpression of other proteins stimulating glucose consumption in the *BRAF/RAS* wild-type population may explain this observation. Potentially relevant proteins include MEK1/2 (8% of melanomas), involved in the MAPK-pathway [25], and mTOR (10.4% of primary melanomas) or PDK1, involved in the PI3K-Akt-mTOR pathway [28, 29]. Furthermore, patient’s *BRAF* status is determined based on one tumour tissue sample, not uncommonly the (excised) primary melanoma, and consequently does not necessarily represent the mutational status of all metastases within a patient [30].

Patients with an elevated serum LDH level—a well-established prognostic biomarker for both worse survival and poor treatment response—had higher tumour ^18^F-FDG uptake as well as higher metabolic tumour volume compared to those with normal LDH levels. However, we also observed tumours with high ^18^F-FDG in patients with (still) relatively low MATV and normal LDH levels. Moreover, several patients with an elevated LDH level had only tumour lesions with relatively low ^18^F-FDG uptake. LDH is a cytoplasmic enzyme that catalyses the interconversion of pyruvate and lactate downstream of glycolysis. A high LDH serum level is generally regarded as a marker of cell damage or necrosis, but the exact source of serum LDH levels is unknown. The biological role of LDH in glucose metabolism has also been suggested as an underlying mechanism and in vitro data suggest differential reliance on aerobic glycolysis and oxidative phosphorylation between patients with normal and elevated serum LDH [3, 13]. Unfortunately, meaningful multivariate approaches to unravel the interrelations between tumour volume, tumour glucose consumption and a proposed metabolic factor underlying serum LDH levels were prohibited by collinearity in our data.

Metabolic targeting may constitute a promising novel approach for patients with tumours with high glucose uptake identified by ^18^F-FDG PET. Furthermore, glycolysis results in extracellular accumulation of lactate and low pH, which are known to impair immune cell function and contribute to an immunosuppressive tumour microenvironment [3, 5]. Metabolic interference combined with immunotherapy might thus be attractive for improving immunotherapy response, for instance in the poorly responding group of metastatic melanoma patients with elevated LDH levels. Metabolic cancer therapies have numerous specific metabolic targets and so far, studies into the correlation between melanoma expression of specific glycolytic transporters and enzymes, such as GLUT1 and hexokinase (HK), and ^18^F-FDG uptake are limited and contradictive [31, 32]. New studies are needed to integrate tumour ^18^F-FDG uptake and other clinical biomarkers with tumour dependence upon specific metabolic pathways and targetable metabolic transporters and enzymes.

Limitations of our study include its retrospective nature and patient heterogeneity in treatment, which allowed only preliminary estimates of the influence of tumour PET parameters on survival. The lack of ce-CT in several patients and its more detailed anatomical lesion information could have resulted in erroneous inclusion of physiological PET-positive lesions or exclusion of malignant PET-positive lesions, respectively. Since tumour measurements were performed on PET images only, necrotic areas (observed in three patients) and brain metastases (*n* = 22) are not incorporated in the MATV.

## Conclusions

Tumour ^18^F-FDG uptake is heterogeneous within and among metastatic melanoma patients. High ^18^F-FDG uptake is independent of *BRAF*/*RAS* mutation status and does not fully correlate with serum LDH levels. This suggests ^18^F-FDG PET metabolic parameters could serve as an (additional) selection tool for melanoma patients potentially benefiting from metabolic therapies. Further investigation of the prognostic and predictive value of quantitative ^18^F-FDG PET parameters is warranted.

## Additional files

Additional file 1: **Figure S1.** Patient selection. Flow diagram showing the selection of eligible patients. (DOCX 547 kb)

Additional file 2: **Table S1.** ^18^F-FDG PET lesion parameters on a per-lesion basis. (DOCX 14 kb)

Additional file 3: **Figure S2.** Number of tumour lesions per metastatic location (A) and total MATV (B) per location. In total, 1143 tumour lesions ≥ 1 ml were identified in 64 patients. The outer ring in (A) displays the distribution when lesions < 1 ml are incorporated as well (total lesion *n* = 3408), showing only minor differences. Total MATV of all 1143 lesions was 14,560 ml (B). LN = lymph node. (DOCX 796 kb)

Additional file 4: **Figure S3.** Individual tumour lesion SUV_peak_ (A) and MATV (B) per metastatic location. SUV_peak_ (A) and MATV (B) of individual lesions ≥ 1 ml (total *n* = 1143), displayed per metastatic location. Boxes represent interquartile range and whiskers respectively 25th and 75th percentile + 1.5 interquartile range. (DOCX 805 kb)

Additional file 5: **Figure S4.** Kaplan-Meier overall survival estimates stratified by LDH levels and PET parameters. Following baseline ^18^F-FDG PET/CT scan, 30 patients (46.9%) started with immunotherapy, 20 patients (31.3%) started with BRAF(/MEK) inhibition, and 5 patients (7.8%) commenced dacarbazine chemotherapy. Nine patients (14.1%) did not receive any systemic treatment. Twenty-one of the included 64 patients (32.8%) were still alive at the time of analysis (17.9 months after the last included baseline PET/CT scan). Curves display overall survival of all patients (*n* = 64) stratified by normal vs. elevated (i.e. > 250 U/l) LDH levels and patient population median of respectively maximum SUV_peak_ (A), total MATV (B) and total TLG (C). LDH = lactate dehydrogenase. (DOCX 271 kb)

## Abbreviations

^18^F-FDG: ^18^F-fluorodeoxyglucose
AJCC: American Joint Committee on Cancer
Akt: Protein kinase B
ce: Contrast-enhanced
CT: Computed tomography
EANM: European Association of Nuclear Medicine
EARL: EANM Research Limited
HK: Hexokinase
IHC: Immunohistochemistry
LD: Low-dose
LDH: Lactate dehydrogenase
MAPK: Mitogen-activated protein kinase
MATV: Metabolically active tumour volume
MEK: Mitogen-activated protein kinase kinase
MRI: Magnetic resonance imaging
mTOR: Mammalian target of rapamycin
PD-L1: Programmed death-ligand 1
PET: Positron emission tomography
PI3K: Phosphoinositide 3-kinase
RANO-BM: Response Assessment in Neuro-Oncology Brain Metastases
ROI: Region of interest
SUV: Standardized uptake value
TLG: Total lesion glycolysis
UMCG: University Medical Center Groningen
VOI: Volume of interest

## References

[1] Ugurel S, Röhmel J, Ascierto PA, Flaherty KT, Grob JJ, Hauschild A (2016). Survival of patients with advanced metastatic melanoma: the impact of novel therapies. Eur J Cancer.

[2] Brand A, Singer K, Koehl GE, Kolitzus M, Schoenhammer G, Thiel A (2016). LDHA-associated lactic acid production blunts tumor immunosurveillance by T and NK cells. Cell Metab.

[3] Blank CU, Haanen JB, Ribas A, Schumacher TN (2016). The “cancer immunogram”. Science.

[4] Vander Heiden MG, Cantley LC, Thompson CB (2009). Understanding the Warburg effect: the metabolic requirements of cell proliferation. Science.

[5] Pavlova NN, Thompson CB (2016). The emerging hallmarks of cancer metabolism. Cell Metab.

[6] Michelakis ED, Sutendra G, Dromparis P, Webster L, Haromy A, Niven E (2010). Metabolic modulation of glioblastoma with dichloroacetate. Sci Transl Med.

[7] Martinez-Outschoorn UE, Peiris-Pagés M, Pestell RG, Sotgia F, Lisanti MP (2017). Cancer metabolism: a therapeutic perspective. Nat Rev Clin Oncol.

[8] Vartanian A, Agnihotri S, Wilson MR, Burrell KE, Tonge PD, Alamsahebpour A (2016). Targeting hexokinase 2 enhances response to radio-chemotherapy in glioblastoma. Oncotarget.

[9] Bénéteau M, Zunino B, Jacquin MA, Meynet O, Chiche J, Pradelli LA (2012). Combination of glycolysis inhibition with chemotherapy results in an antitumor immune response. Proc Natl Acad Sci U S A.

[10] Ganapathy-Kanniappan S, Geschwind JF (2013). Tumor glycolysis as a target for cancer therapy: progress and prospects. Mol Cancer.

[11] Garon EB, Christofk HR, Hosmer W, Britten CD, Bahng A, Crabtree MJ (2014). Dichloroacetate should be considered with platinum-based chemotherapy in hypoxic tumors rather than as a single agent in advanced non-small cell lung cancer. J Cancer Res Clin Oncol.

[12] Shestov AA, Mancuso A, Lee SC, Guo L, Nelson DS, Roman JC (2016). Bonded cumomer analysis of human melanoma metabolism monitored by ^13^C NMR spectroscopy of perfused tumor cells. J Biol Chem.

[13] Ho J, de Moura MB, Lin Y, Vincent G, Thorne S, Duncan LM (2012). Importance of glycolysis and oxidative phosphorylation in advanced melanoma. Mol Cancer.

[14] Balch CM, Gershenwald JE, Soong SJ, Thompson JF, Atkins MB, Byrd DR (2009). Final version of 2009 AJCC melanoma staging and classification. J Clin Oncol.

[15] Boellaard R, Delgado-Bolton R, Oyen WJ, Giammarile F, Tatsch K, Eschner W (2015). FDG PET/CT: EANM procedure guidelines for tumour imaging: version 2.0. Eur J Nucl Med Mol Imaging.

[16] Strobel K, Dummer R, Husarik DB, Pérez Lago M, Hany TF, Steinert HC (2007). High-risk melanoma: accuracy of FDG PET/CT with added CT morphologic information for detection of metastases. Radiology.

[17] Lin NU, Lee EQ, Aoyama H, Barani IJ, Barboriak DP, Baumert BG (2015). Response assessment criteria for brain metastases: proposal from the RANO group. Lancet Oncol.

[18] Ilie M, Khambata-Ford S, Copie-Bergman C, Huang L, Juco J, Hofman V (2017). Use of the 22C3 anti–PD-L1 antibody to determine PD-L1 expression in multiple automated immunohistochemistry platforms. PLoS One.

[19] Schoenewolf NL, Belloni B, Simcock M, Tonolla S, Vogt P, Scherrer E (2014). Clinical implications of distinct metastasizing preferences of different melanoma subtypes. Eur J Dermatology.

[20] Frauchiger AL, Mangana J, Rechsteiner M, Moch H, Seifert B, Braun RP (2016). Prognostic relevance of lactate dehydrogenase and serum S100 levels in stage IV melanoma with known BRAF mutation status. Br J Dermatol.

[21] Hall A, Meyle KD, Lange MK, Klima M, Sanderhoff M, Dahl C (2013). Dysfunctional oxidative phosphorylation makes malignant melanoma cells addicted to glycolysis driven by the (V600E)BRAF oncogene. Oncotarget.

[22] Hardeman KN, Peng C, Paudel BB, Meyer CT, Luong T, Tyson DR (2017). Dependence on glycolysis sensitizes BRAF-mutated melanomas for increased response to targeted BRAF inhibition. Sci Rep.

[23] Nazarian R, Shi H, Wang Q, Kong X, Koya RC, Lee H (2010). Melanomas acquire resistance to B-RAF(V600E) inhibition by RTK or N-RAS upregulation. Nature.

[24] Kerr EM, Gaude E, Turrell FK, Frezza C, Martins CP (2016). Mutant Kras copy number defines metabolic reprogramming and therapeutic susceptibilities. Nature.

[25] Richtig G, Hoeller C, Kashofer K, Aigelsreiter A, Heinemann A, Kwong LN (2017). Beyond the BRAF^V600E^ hotspot: biology and clinical implications of rare BRAF gene mutations in melanoma patients. Br J Dermatol.

[26] Choi EK, Chong A, Ha JM, Jung CK, O JH, Kim SH (2017). Clinicopathological characteristics including BRAF V600E mutation status and PET/CT findings in papillary thyroid carcinoma. Clin Endocrinol.

[27] Yoon M, Jung SJ, Kim TH, Ha TK, Urm SH, Park JS (2016). Relationships between transporter expression and the status of BRAF V600E mutation and F-18 FDG uptake in papillary thyroid carcinomas. Endocr Res.

[28] Yan K, Si L, Li Y, Wu X, Xu X, Dai J (2016). Analysis of mTOR gene aberrations in melanoma patients and evaluation of their sensitivity to PI3K-AKT-mTOR pathway inhibitors. Clin Cancer Res.

[29] Pópulo H, Caldas R, Lopes JM, Pardal J, Máximo V, Soares P (2015). Overexpression of pyruvate dehydrogenase kinase supports dichloroacetate as a candidate for cutaneous melanoma therapy. Expert Opin Ther Targets.

[30] Riveiro-Falkenbach E, Santos-Briz A, Ríos-Martín JJ, Rodríguez-Peralto JL (2017). Controversies in intrapatient melanoma BRAF^V600E^ mutation status. Am J Dermatopathol.

[31] Park SG, Lee JH, Lee WA, Han KM (2012). Biologic correlation between glucose transporters, hexokinase-II, Ki-67 and FDG uptake in malignant melanoma. Nucl Med Biol.

[32] Yamada K, Brink I, Bissé E, Epting T, Engelhardt R (2005). Factors influencing [F-18] 2-fluoro-2-deoxy-D-glucose (F-18 FDG) uptake in melanoma cells: the role of proliferation rate, viability, glucose transporter expression and hexokinase activity. J Dermatol.

