# Supplementary material for: Mapping heterogeneity in glucose uptake in metastatic melanoma using quantitative 18F-FDG PET/CT analysis
Source: EJNMMI Res. 2018 Nov 20;8:101. doi: 10.1186/s13550-018-0453-x (PMC6246760; doi:10.1186/s13550-018-0453-x)
Supplement: Supplementary file 1 — Figure S1. Patient selection. Flow diagram showing the selection of eligible patients. (DOCX 547 kb) [file 13550_2018_453_MOESM1_ESM.docx]

##
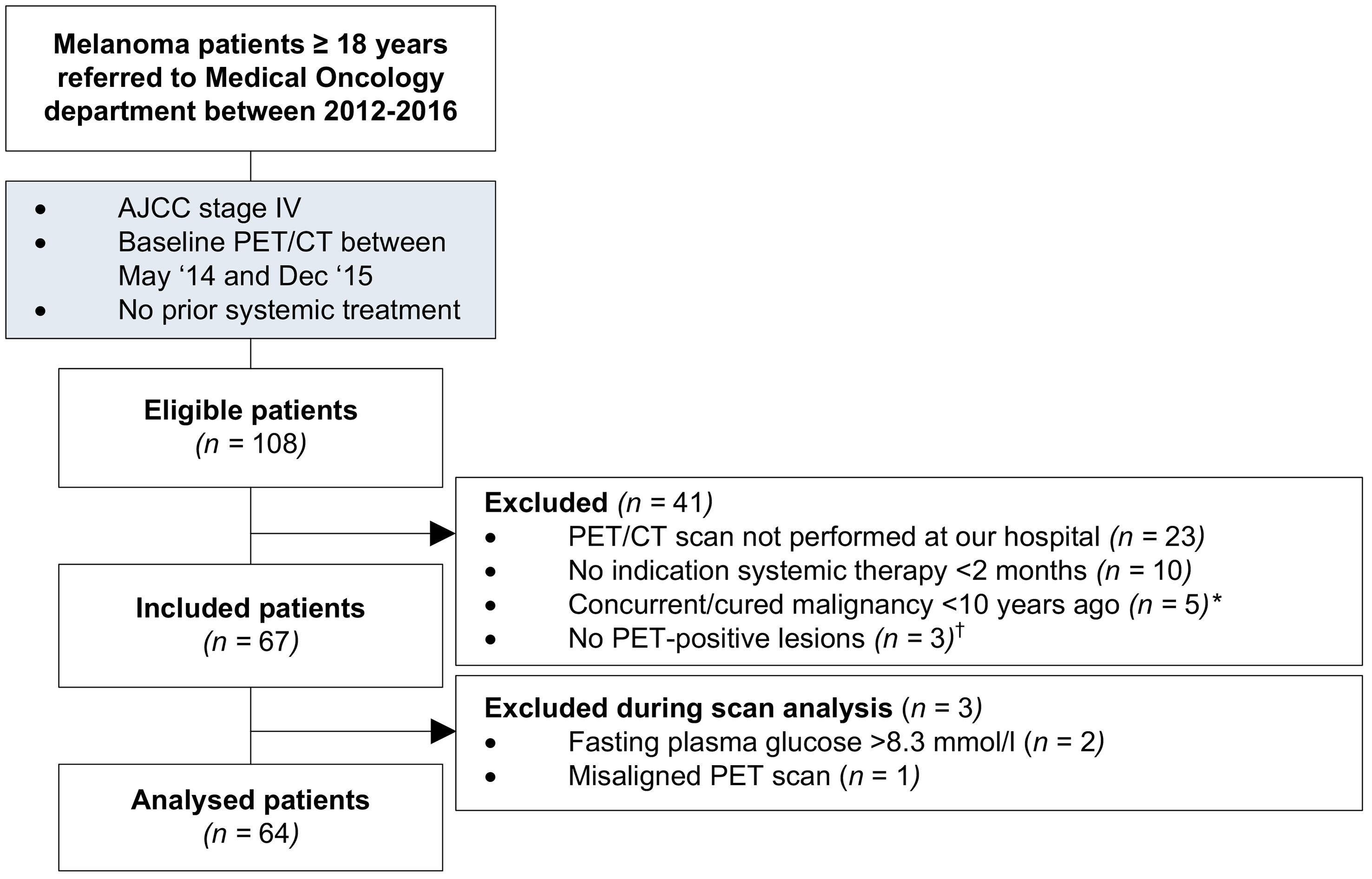


**Figure S1** Patient selection. Flow diagram showing selection of eligible patients.

* One patient with active pulmonal lymphangioleiomyomatosis was excluded based on contradictive literature on differentiating lymphangioleiomyomatosis from tumor lesions on ^18^F-FDG PET/CT.

† One patient with PET-negative diffuse bone marrow metastases (biopsy-confirmed) and two patients with only brain metastases.

AJCC = American Joint Committee on Cancer
