# Supplementary material for: Mapping heterogeneity in glucose uptake in metastatic melanoma using quantitative 18F-FDG PET/CT analysis
Source: EJNMMI Res. 2018 Nov 20;8:101. doi: 10.1186/s13550-018-0453-x (PMC6246760; doi:10.1186/s13550-018-0453-x)
Supplement: Supplementary file 2 — Table S1. 18F-FDG PET lesion parameters on a per-lesion basis. (DOCX 14 kb) [file 13550_2018_453_MOESM2_ESM.docx]

**Table S1** ^18^F-FDG PET lesion parameters on a per-lesion basis.

|  | **All lesions (*n* = 1143)** | |
| --- | --- | --- |
|  |  | *range* |
| **SUV_peak_** | 5.0 (3.4-7.8) | 0.7-58.3 |
| **SUV_max_** | 6.8 (4.6-10.7) | 1.1-67.2 |
| **SUV_mean_** | 4.6 (3.4-6.8) | 0.7-30.2 |
| **MATV (ml)** | 2.4 (1.4-6.1) | 1.0-1921 |
| **TLG** | 11.4 (5.8-34.0) | 1.1-11206 |

Data are displayed as median (interquartile range)
