# Supplementary material for: Mapping heterogeneity in glucose uptake in metastatic melanoma using quantitative 18F-FDG PET/CT analysis
Source: EJNMMI Res. 2018 Nov 20;8:101. doi: 10.1186/s13550-018-0453-x (PMC6246760; doi:10.1186/s13550-018-0453-x)
Supplement: Supplementary file 3 — Figure S2. Number of tumour lesions per metastatic location (A) and total MATV (B) per location. In total, 1143 tumour lesions ≥ 1 ml were identified in 64 patients. The outer ring in (A) displays the distribution when lesions < 1 ml are incorporated as well (total lesion n = 3408), showing only minor differences. Total MATV of all 1143 lesions was 14,560 ml (B). LN = lymph node. (DOCX 796 kb) [file 13550_2018_453_MOESM3_ESM.docx]

###
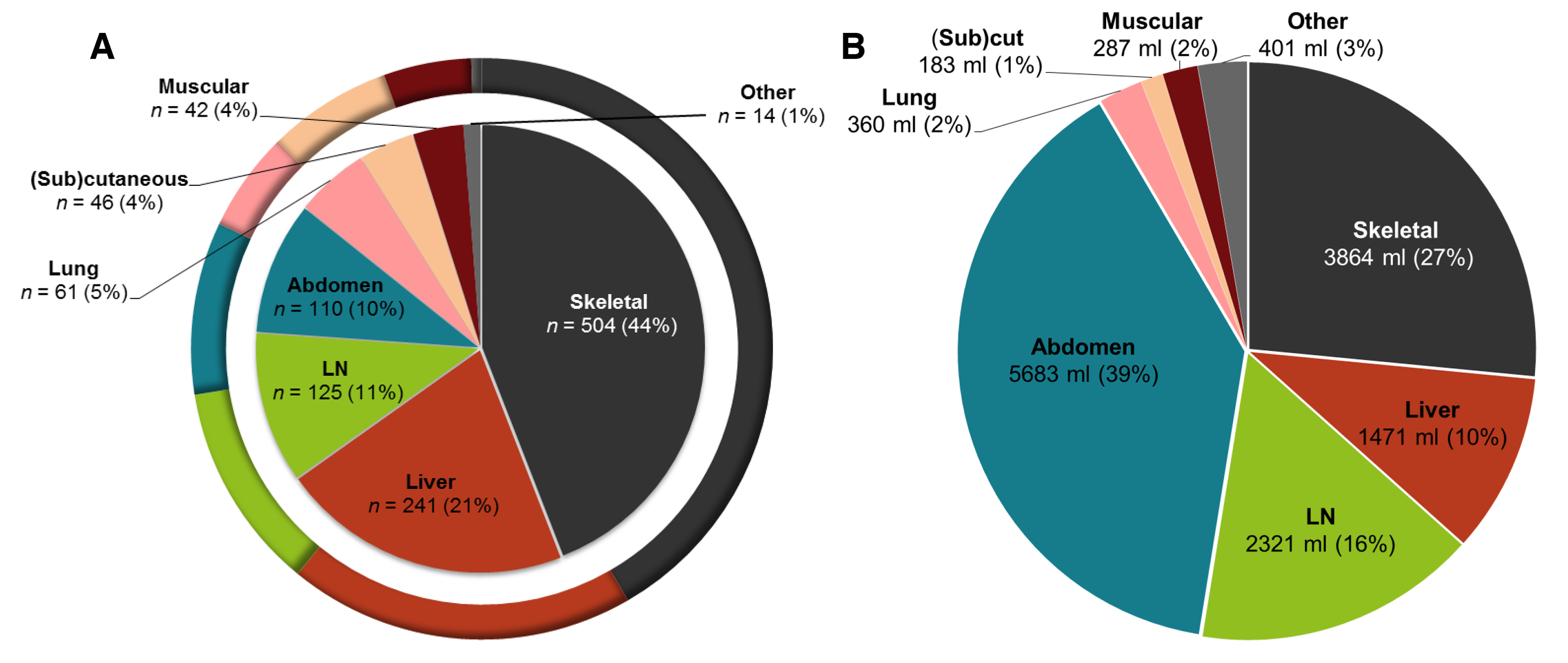
Figure S2 Number of tumor lesions per metastatic location (A) and total MATV (B) per location. In total, 1143 tumor lesions ≥1 ml were identified in 64 patients. The outer ring in (A) displays the distribution when lesions <1 ml are incorporated as well (total lesion *n* = 3408), showing only minor differences. Total MATV of all 1143 lesions was 14560 ml (B) LN = lymph node
