# Supplementary material for: Mapping heterogeneity in glucose uptake in metastatic melanoma using quantitative 18F-FDG PET/CT analysis
Source: EJNMMI Res. 2018 Nov 20;8:101. doi: 10.1186/s13550-018-0453-x (PMC6246760; doi:10.1186/s13550-018-0453-x)
Supplement: Supplementary file 4 — Figure S3. Individual tumour lesion SUVpeak (A) and MATV (B) per metastatic location. SUVpeak (A) and MATV (B) of individual lesions ≥ 1 ml (total n = 1143), displayed per metastatic location. Boxes represent interquartile range and whiskers respectively 25th and 75th percentile + 1.5 interquartile range. (DOCX 805 kb) [file 13550_2018_453_MOESM4_ESM.docx]

**
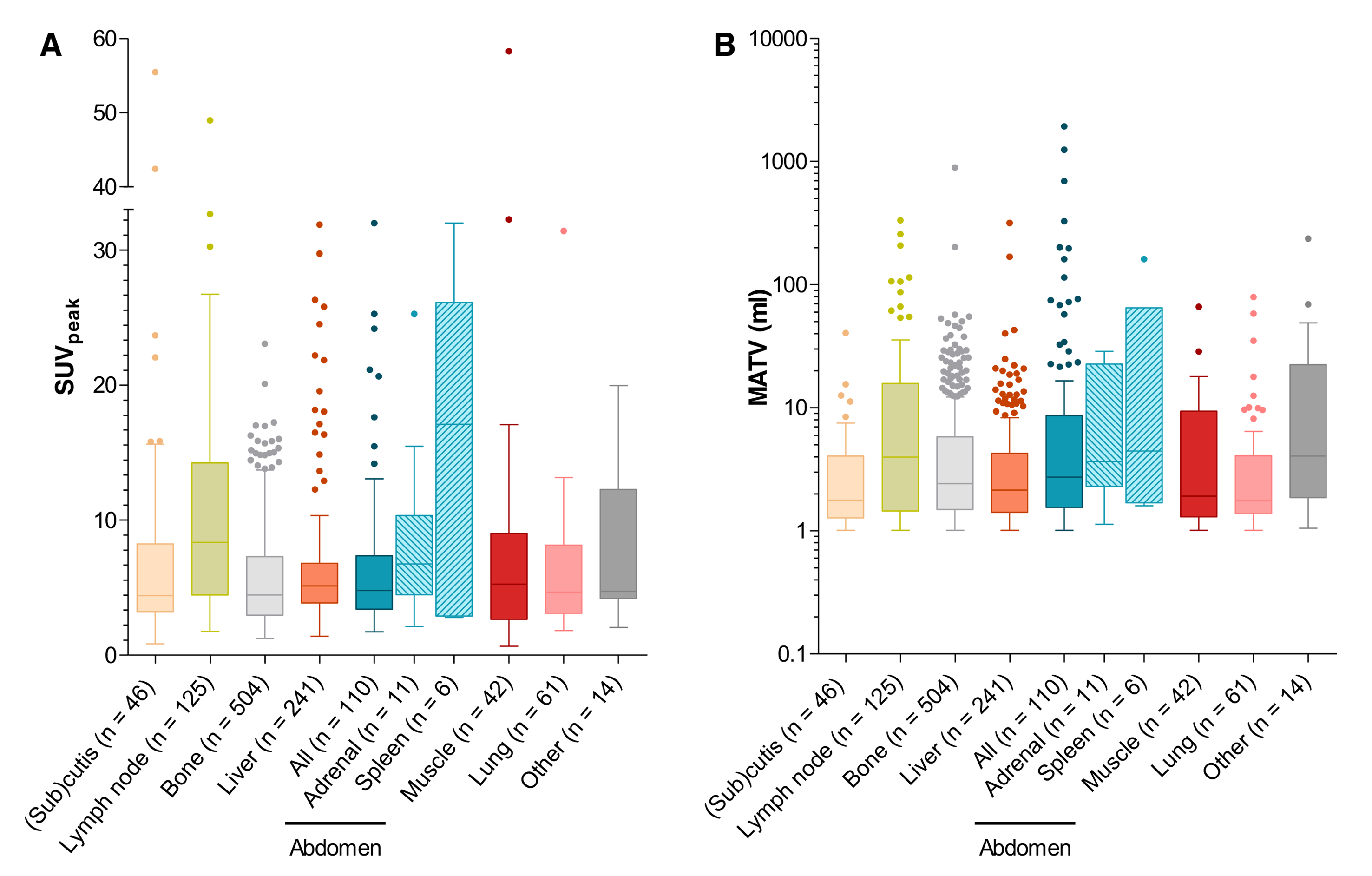
**

**Figure S3** Individual tumor lesion SUV_peak_ (A) and MATV (B) per metastatic location. SUV_peak_ (A) and MATV (B) of individual lesions ≥1 ml (total *n* = 1143), displayed per metastatic location. Boxes represent interquartile range and whiskers respectively 25^th^ and 75^th^ percentile +1.5 interquartile range.
