# Supplementary material for: Mapping heterogeneity in glucose uptake in metastatic melanoma using quantitative 18F-FDG PET/CT analysis
Source: EJNMMI Res. 2018 Nov 20;8:101. doi: 10.1186/s13550-018-0453-x (PMC6246760; doi:10.1186/s13550-018-0453-x)
Supplement: Supplementary file 5 — Figure S4. Kaplan-Meier overall survival estimates stratified by LDH levels and PET parameters. Following baseline 18F-FDG PET/CT scan, 30 patients (46.9%) started with immunotherapy, 20 patients (31.3%) started with BRAF(/MEK) inhibition, and 5 patients (7.8%) commenced dacarbazine chemotherapy. Nine patients (14.1%) did not receive any systemic treatment. Twenty-one of the included 64 patients (32.8%) were still alive at the time of analysis (17.9 months after the last included baseline PET/CT scan). Curves display overall survival of all patients (n = 64) stratified by normal vs. elevated (i.e. > 250 U/l) LDH levels and patient population median of respectively maximum SUVpeak (A), total MATV (B) and total TLG (C). LDH = lactate dehydrogenase. (DOCX 271 kb) [file 13550_2018_453_MOESM5_ESM.docx]

*
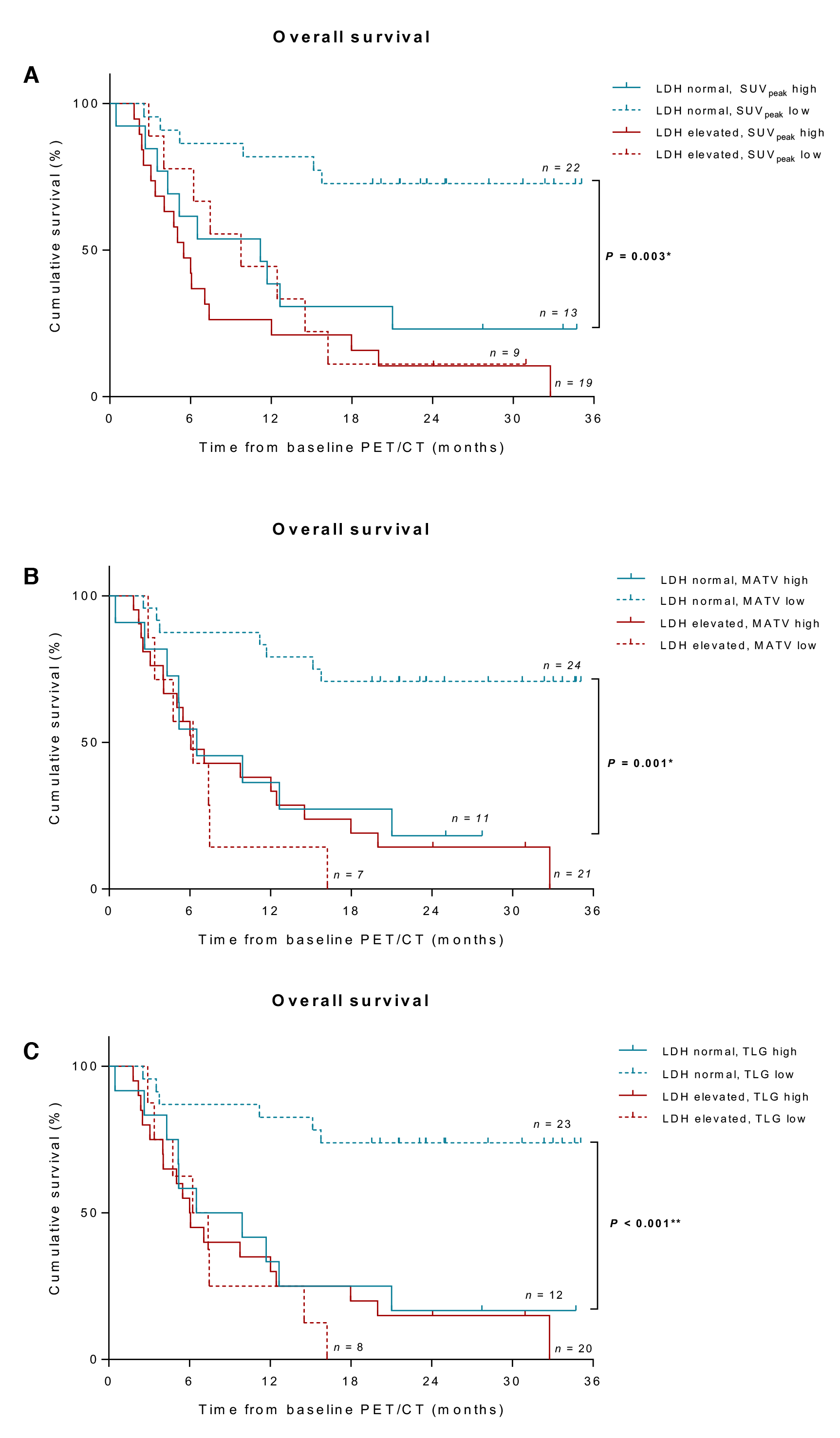
*

**Figure S4** Kaplan-Meier overall survival estimates stratified by LDH levels and PET parameters. Following baseline ^18^F-FDG PET/CT scan, 30 patients (46.9%) started with immunotherapy, 20 patients (31.3%) started with BRAF(/MEK) inhibition, and five patients (7.8%) commenced dacarbazine chemotherapy. Nine patients (14.1%) did not receive any systemic treatment. 21 of the included 64 patients (32.8%) were still alive at time of analysis (17.9 months after the last included baseline PET/CT scan). Curves display overall survival of all patients (*n* = 64) stratified by normal vs. elevated (i.e. >250 U/l) LDH levels and patient population median of respectively maximum SUV_peak_ (A), total MATV (B) and total TLG (C). LDH = lactate dehydrogenase
